# Efficacy and safety of xyloglucan nasal spray in children with allergic rhinitis: A randomized, double-blind, placebo-controlled, crossover study

**DOI:** 10.5415/apallergy.0000000000000221

**Published:** 2025-08-13

**Authors:** Piyapong Laopakorn, Potjanee Kiewngam, Wanlapa Jotikasthira, Adithep Sawatchai, Wacharoot Kanchongkittiphon, Wiparat Manuyakorn

**Affiliations:** 1 Division of Allergy and Immunology, Department of Pediatrics, Ramathibodi Hospital, Mahidol University, Bangkok, Thailand

**Keywords:** A tamarind seed extract-based saline solution, daily symptom score, mucosal barrier, nonpharmacological treatment, pediatric allergy, visual analog scale

## Abstract

**Background:**

A tamarind seed extract-based solution containing xyloglucan forms a protective layer over the nasal epithelium. However, there is no previous study evaluating xyloglucan efficacy in children with allergic rhinitis (AR).

**Objective:**

To evaluate the safety and efficacy of xyloglucan as an adjunctive treatment for AR in children.

**Methods:**

This randomized, double-blind, placebo-controlled crossover study assessed the efficacy of xyloglucan nasal spray in managing AR symptoms in children. Participants 6–18 years with AR were randomized to receive either xyloglucan or placebo in addition to their current AR therapy for 2 weeks, followed by a 2-week washout and crossover to the alternate treatment for 2 weeks. Primary outcomes were changes in visual analog scale scores analyzed using linear mixed models, adjusting for treatment, period, sequence, and compliance. Subgroup analyses included children with moderate symptoms and good compliance.

**Results:**

Seventy-nine children with AR were enrolled, median age 10 years (IQR: 8–12). Treatment with xyloglucan was associated with a significantly greater reduction in nasal congestion compared to placebo (β = −1.013, 95% confidence interval [CI], −1.729 to −0.298, *P* = 0.006). In children with moderate symptoms and good compliance, the effect on nasal congestion was more pronounced (β = −1.588, *P* = 0.046). Improvements in rhinorrhea, sneezing, and itching were observed but did not reach statistical significance. Both treatments were well tolerated, with only mild adverse events in both groups.

**Conclusion:**

Xyloglucan nasal spray, as an adjunct to standard AR treatment, demonstrated potential benefits in alleviating rhinitis symptoms, particularly nasal congestion, in children with AR. This clinical trial was registered with the Thai Clinical Trials Registry (No. TCTR20240318004).

## 1. Introduction

Allergic rhinitis (AR) is an upper airway disease characterized by clinical symptoms, including congestion, sneezing, rhinorrhea, and nasal itching. AR is associated with significant morbidity, poor quality of life, and considerable healthcare expenses [1]. Treatments for AR include allergen avoidance and medical interventions, such as antihistamines and intranasal corticosteroids (INCs). INCs are considered the first-line therapy due to their anti-inflammatory effects on mucosal inflammation [2]. Despite numerous studies on the safety of INCs, they can have side effects, including epistaxis. Additionally, several studies have shown adverse effects on growth when using high doses of INCs [3]. As a result, alternative treatments, such as the mucosal epithelial barriers, have been explored [4, 5].

Disruption of the epithelial barrier function, resulting in exposure to allergens, leads to the development of several allergic diseases, including AR [6]. Nonpharmacological approaches to preventing epithelial barrier disruption and creating a mechanical barrier over the mucosa could reduce contact with allergens, irritants, pathogens, and triggering factors [7]. Xyloglucan, a neutral, branched polysaccharide, consists of a cellulose-like backbone extracted from tamarind seed. The configuration of this polysaccharide results in a mucin-like molecular product with optimal mucoadhesive properties, acting as a physical barrier that protects different agents, such as microorganisms, allergens, and pro-inflammatory compounds [7]. Xyloglucan has been shown to prevent the interaction between the mucosa and harmful agents, including allergens, bacteria, and inflammatory stimuli, without systemic absorption or pharmacological interference [7]. In a murine AR model, intranasal xyloglucan reduced nasal symptoms, eosinophilic inflammation, and cytokines (interleukin [IL]-4, IL-5, and IL-13), showing efficacy comparable to INCs [2]. It also preserved ZO-1 expression and reduced epithelial permeability, indicating a role in barrier stabilization [2]. A previous study has suggested that xyloglucan can reduce the nasal symptom scores in adult patients with moderate to severe symptoms of rhinosinusitis [8]. However, the efficacy of xyloglucan in controlling AR symptoms in children remains scarce. The current study evaluated the efficacy of xyloglucan nasal spray in controlling symptoms of AR in children.

## 2. Methods

This randomized, double-blind, placebo-controlled crossover study was conducted between June 2023 and April 2024. The Human Rights and Ethics Committee reviewed and approved the study (MURA2023/137). All participants and their parents provided their written informed consent. This clinical trial was registered with the Thai Clinical Trials Registry (No. TCTR20240318004). This study was reported in accordance with the CONSORT 2010 guidelines and the CONSORT 2019 extension for randomized crossover trials [9]. The design, conduct, analysis, and reporting of the crossover features—including randomization, period effects, treatment sequence, washout, and intrasubject comparisons—were conducted to ensure methodological rigor and transparency. A completed CONSORT crossover checklist is included as Supplementary Material https://links.lww.com/PA9/A67.

### 2.1 Study population

The study enrolled children aged 6–18 years with AR who showed aeroallergen sensitization by skin prick test or specific immunoglobulin E and received daily INC treatment for at least 4 consecutive weeks, but continued to experience persistent symptoms. The details of dose and type of INC treatment were summarized in Table 1. Exclusion criteria included (1) comorbid cardiovascular disease or primary immunodeficiency, (2) active tuberculosis infection, (3) structural upper and lower airway anomalies, (4) receiving allergen immunotherapy, (5) recent respiratory tract infection within 4 weeks, and (6) pregnancy or breastfeeding.

**Table 1.** Demographics and baseline characteristics

Characteristics and variables	N = 79
Age (year) (median [IQR])	10 (8, 12)
Sex male (n [%])	53 (67.1)
Underlying disease (n [%])	41 (51.9)
• Asthma	19 (24.1)
• Atopic dermatitis	16 (20.3)
• Food allergy	4 (5.1)
Skin prick test (n [%])	
• HDM	71 (89.9)
• Cat	20 (25.3)
• Cockroach	18 (22.8)
• Dog	13 (16.5)
• Grass	8 (10.1)
Treatment (n [%])	
• Intranasal steroid	79 (100)
-Mometasone/Fluticasone furoate	75 (94.9)
1 puff OD	23 (30.7)
2 puff OD	51 (68)
2 puff BID	1 (1.3)
-Fluticasone/Azelastine	4 (5.1)
• Oral antihistamine	79 (100)
• Oral LTRA	7 (8.9)
Baseline VAS: median (IQR)	4 (3,6)
• VAS < 5 (n [%])	38 (48.1)
• VAS ≥ 5 (n [%])	41 (51.9)
Good compliance* (n [%])	
• Xyloglucan	59 (74.7)
• Placebo	55 (69.6)

BID, twice a day; HDM, house dust mite; LTRA, leukotriene receptor antagonist; OD, once a day; VAS, visual analog scales.

* Define by using spray more than 80% measure by study drug weight.

### 2.2 Study protocol

**Figure 1.**
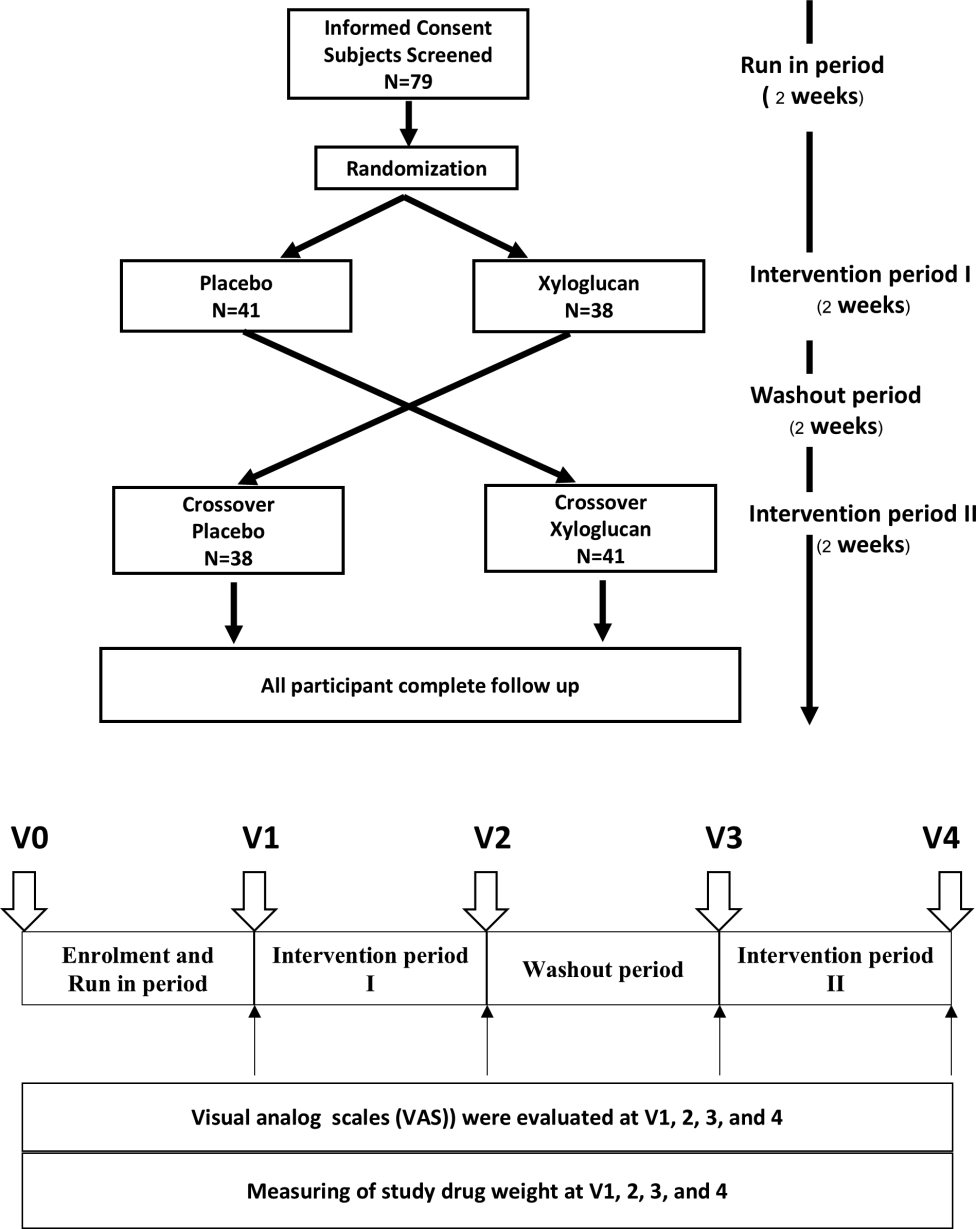
Study protocol and subject disposition.

Baseline characteristics were recorded at screening, including medical history, aeroallergen sensitization, and current medication. Visual analog scales (VAS) were evaluated at baseline and after treatment. The VAS is a 10 cm line grading the severity of symptoms from 0 = absent to 10 = highest level and was recorded for each of the 4 symptoms, including nasal congestion, itching, rhinorrhea, and sneezing [10]. Participants continued using their current rhinitis medication, including INCs or oral antihistamines. The number of days on which rescue medications were used—including pseudoephedrine, additional doses of oral antihistamines, or INCs—was recorded from the patients’ daily medication record forms. After the 2-week run-in periods, all participants were randomized to initially receive either tamarind seed extract-based saline solution (xyloglucan) or placebo, each administered as 2 sprays per nostril 3 times daily (12 sprays per day in total) for 2 weeks as adjunctive therapy to their current intranasal steroid treatment (Fig. 1). The randomization lists were generated in blocks of 4 using a sealed envelope tool from sealedenvelope.com. The placebo had the same ingredients as the study drug except for the active ingredient, xyloglucan. Placebo also had the same particle size and appearance as the xyloglucan spray. Xyloglucan and placebo were supplied by DEVINTEC PHARMA, Italy. The spray devices containing the 2 study medications were physically and smelled indistinguishable and labeled with serial numbers. Both participants and investigators were blinded to randomization codes. Blinding codes were kept until all participants completed the study. The study evaluated drug compliance by measuring drug weight. Good compliance was defined as using a study drug weight of more than 80%. Text messages were sent twice daily to remind participants to use the spray.

Primary outcomes were the changes in VAS on rhinitis symptoms compared between post-treatment and the pretreatment score, as calculated by the post-treatment score minus the pretreatment score. Secondary outcomes included (1) adverse events compared between groups and (2) the rescue medication used.

### 2.3 Statistical analysis

All analyses were performed using SPSS version 18 and Python 3.11 (statsmodels and matplotlib/seaborn). Nonparametric tests included the Wilcoxon signed-rank test for within-group medians, the Mann-Whitney *U* test for between-group comparisons, the Chi-square test for frequency differences, and McNemar’s test for adverse event associations. The primary outcomes—VAS scores changes for nasal congestion, rhinorrhea, itching, and sneezing—were analyzed using linear mixed models (LMMs) to account for the crossover design and repeated measures. Each LMM included fixed effects for treatment (xyloglucan vs placebo), period, sequence, and compliance (good vs poor), with a random intercept for each participant. Subgroup analyses were conducted in patients with moderate symptoms (baseline VAS ≥5) and good compliance, using separate LMMs per symptom. Results are presented as beta coefficients (β) with 95% confidence intervals (CIs) and *P*-values. Binary outcomes, such as rescue medication use, were evaluated using generalized linear models (GLMs) with binomial distribution and logit link, adjusting for treatment, sequence, period, and compliance. Odds ratios (ORs) and 95% CIs were reported. Forest plots were generated to visualize treatment effects. A 2-sided *P*-value < 0.05 was considered statistically significant. The sample size (n = 29 per arm) was calculated to detect differences in total nasal symptom scores with 80% power, based on prior data [8].

### 2.4 Use of artificial intelligence tools

Artificial intelligence (AI) tools were employed in specific aspects of manuscript preparation and data analysis. The statistical analyses, including the implementation of LMMs, GLMs, and the generation of forest, were conducted using Python version 3.11 with the assistance of AI-based code generation provided by OpenAI’s ChatGPT-4. This tool was also used to assist in English proofreading of the manuscript. All AI-generated content was critically reviewed and revised for accuracy, scientific integrity, and adherence to journal standards. No data or interpretations were accepted without thorough verification by the authors.

## 3. Results

**Figure 2.**
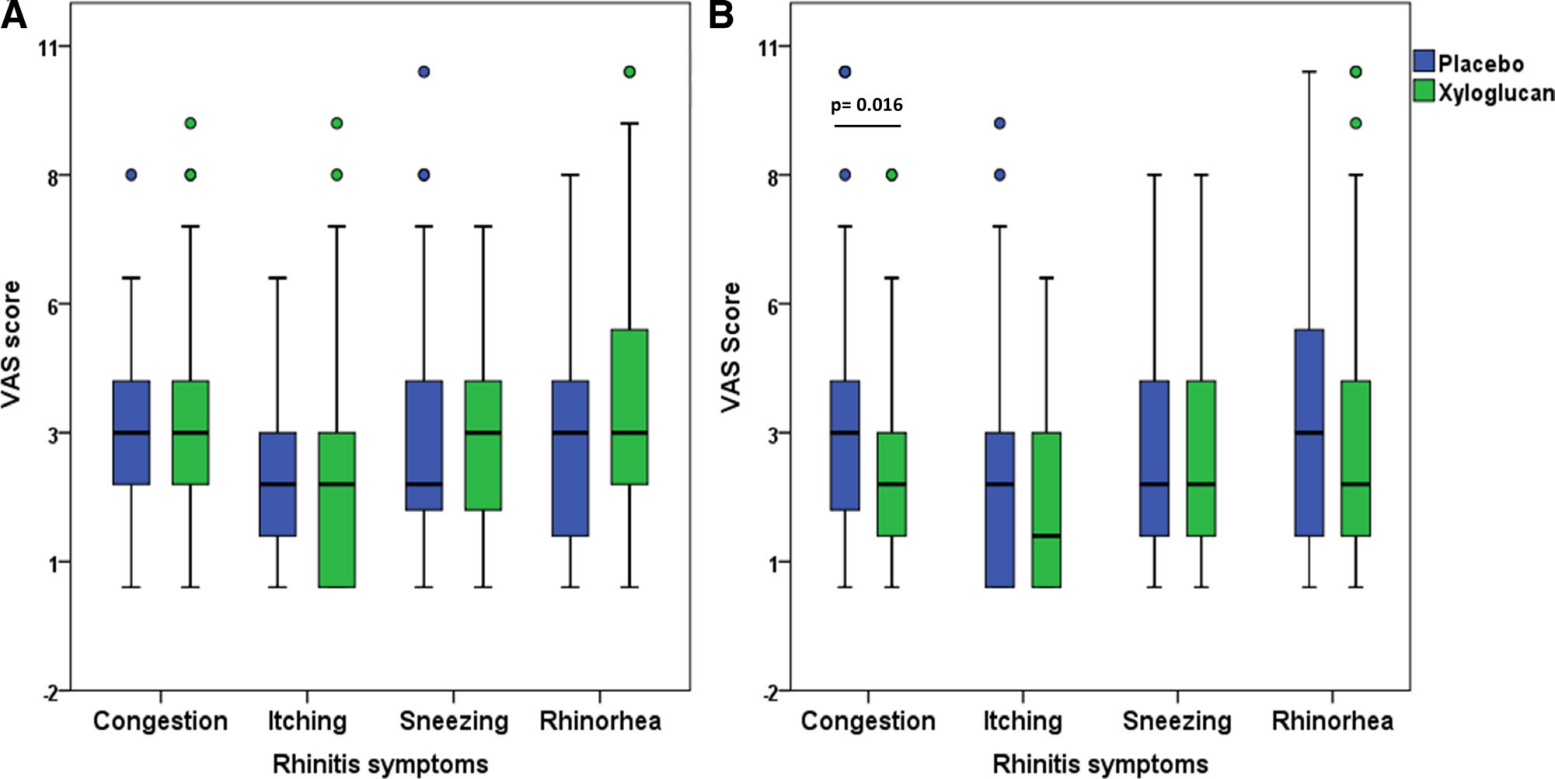
Comparison between VAS in children receiving xyloglucan and placebo. (A) Baseline. (B) After treatment in all enrolled children. VAS, visual analog scales.

A total of 79 children with AR were enrolled, and all enrolled children completed the study protocol (Fig. 1). The median (IQR) age of the enrolled children was 10 (8–12) years, and 53 children (67%) were male. All participants used INCs and antihistamines as their current medication. The baseline characteristics of the enrolled participants are summarized (Table 1). There were no significant differences in the baseline VAS between children initially receiving xyloglucan, then placebo, and placebo then xyloglucan (Table 2). No significant differences in the baseline VAS between children receiving xyloglucan and placebo were demonstrated (Fig. 2A). The overall drug use compliance was 72%. There were no significant differences in drug use compliance when using the xyloglucan or placebo (74.7% vs 69.6%, *P* = 0.25).

**Table 2.** Comparisons of baseline visual analog scales between groups classified by treatment sequence

Variables	All participants (n = 79)		*P*-value
	Sequence placebo-xyloglucan (n = 41)	Sequence xyloglucan-placebo (n = 38)	
Baseline VAS			
-Congestion	3 (2, 4)	3 (2, 6.25)	0.32
-Itching	2 (1, 3.5)	2 (0.75, 5)	0.86
-Sneezing	3 (2, 5)	3 (2, 4.25)	0.63
-Rhinorrhea	3 (2, 4)	4 (2, 6)	0.33

Values are presented as median (IQR). *P*-value corresponds to the Mann-Whitney *U* test for the analysis between the groups.

VAS, visual analogue scale.

### 3.1 Changes in rhinitis symptoms after treatment

In comparison, post-treatment rhinitis symptoms, the VAS on congestion of children while receiving xyloglucan was significantly lower than the VAS on congestion while receiving a placebo (2 [1–3] vs 3 [2–4], *P* = 0.016). However, VAS on itching, sneezing, and rhinorrhea were lower in children receiving xyloglucan, but these failed to reach statistical significance (Fig. 2B).

**Figure 3.**
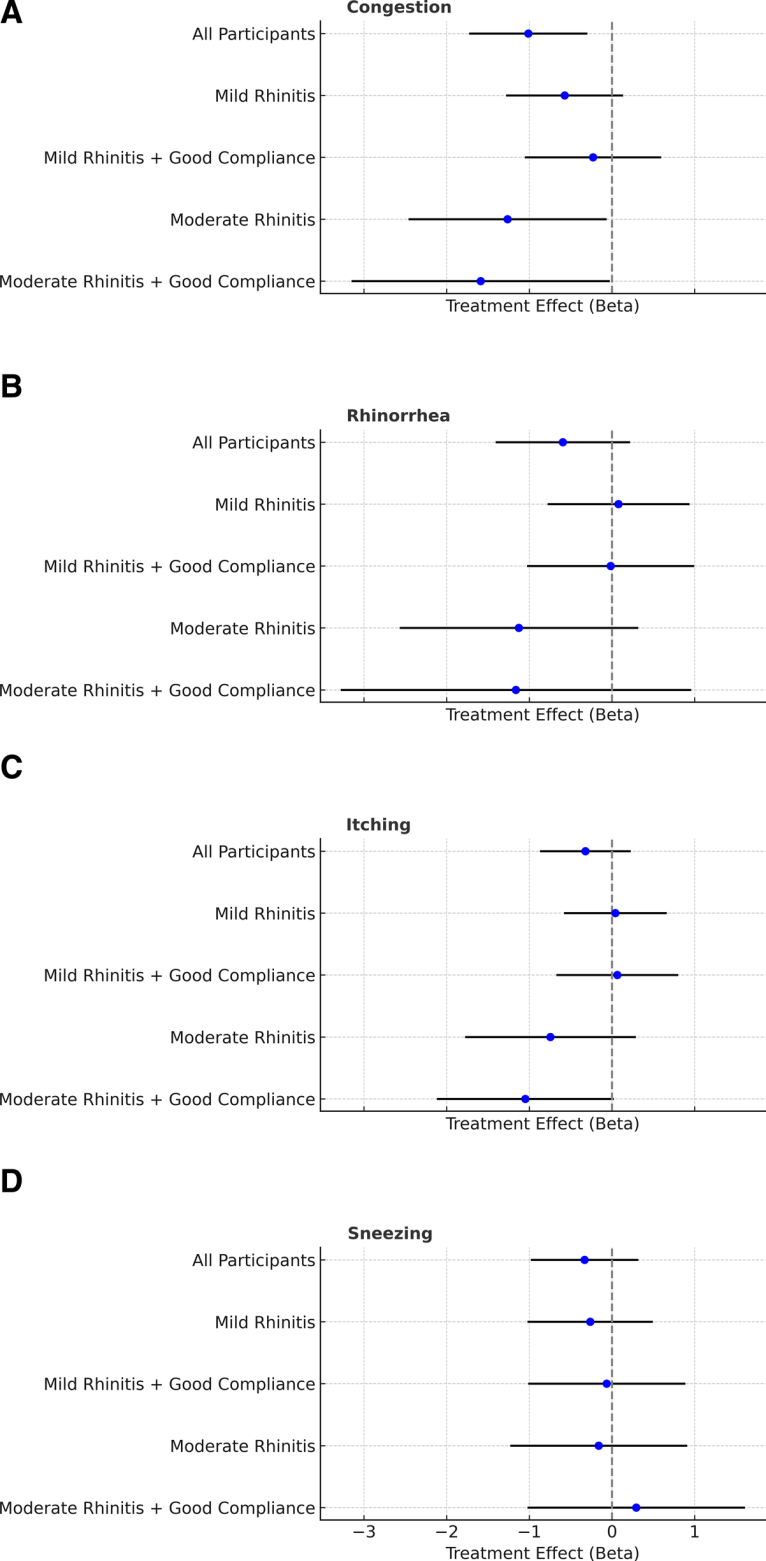
Forest plots of treatment effects (β coefficients and 95% confidence intervals) for xyloglucan nasal spray versus placebo across rhinitis symptoms and subgroups. (A) Nasal congestion. (B) Rhinorrhea. (C) Itching. (D) Sneezing.

LMMs adjusting for treatment, period, and sequence revealed that xyloglucan significantly improved nasal congestion compared to placebo in the overall cohort (β = −1.013; 95% CI, −1.729 to −0.298; *P* = 0.006). In subgroup analysis. Statistically significant improvements were also observed in children with moderate rhinitis, both in those with good compliance (β = −1.588, *P* = 0.046) and the general subgroup (β = −1.264, *P* = 0.039). In contrast, mild rhinitis subgroups demonstrated numerically smaller and nonsignificant improvements (Fig. 3A).

For other rhinitis symptoms—rhinorrhea, itching, and sneezing—xyloglucan showed consistent trends toward improvement, especially in moderate rhinitis subgroups, but none of these reached statistical significance (Fig. 3B–D).

### 3.2 Change in rescue medication use

Rescue medication use was observed in 12% of participants in the xyloglucan group and 15% in the placebo group, with no statistically significant difference between the groups (*P* = 0.646). The mean (SD) number of days on which rescue medications were used over a 2-week period was 4.9 (3.5) days in the xyloglucan group and 4.48 (2.38) days in the placebo. A GLM using a binomial distribution showed that the odds of rescue medication use were lower in the xyloglucan group compared to placebo (OR = 0.799; 95% CI, 0.304–2.102; *P* = 0.650), although this difference was not statistically significant. Inclusion of compliance in the model showed a similar nonsignificant reduction in rescue medication use for those with good compliance (OR = 0.807; 95% CI, 0.301–2.165; *P* = 0.671). These results suggest potential clinical benefit but require further study with larger samples.

### 3.3 Safety and tolerability

Both treatments were well tolerated. More than half of both groups reported adverse events (39 patients in the xyloglucan group and 45 patients in the placebo group). Most adverse events were mild, including sneezing, rhinorrhea, irritation, dry cough, lack of sleep, sore throat, and abnormal taste. Multiple side effects were reported by 20 patients (51%) in the xyloglucan group and 29 patients (64%) in the placebo group. No significant differences were observed in adverse events reported between groups. There were no serious adverse events, and no study drug withdrawal occurred.

## 4. Discussion

The current prospective randomized, double-blind, placebo-controlled crossover study evaluated the efficacy of tamarind seed extract-based saline solution, namely xyloglucan, as an adjunctive therapy to the standard treatment of aeroallergen-sensitized AR children. Our findings show that using xyloglucan nasal spray improves rhinitis symptoms, especially congestion symptoms, as assessed by changes in VAS, compared to a placebo. This study adds to the potential role of mucosal barrier properties for xyloglucan [7] as an adjunctive therapeutic option for managing AR in pediatric populations, since there has been an increase in the prevalence of using complementary and alternative medicine for AR symptoms [11].

Our results align with previous research on xyloglucan nasal spray in adults [8]. Allerini et al. demonstrated that xyloglucan nasal spray treatment for 2 weeks effectively decreased total nasal symptom scores in adults with rhinosinusitis, showing a significant improvement over saline placebo treatments [8]. Moreover, a study in a mouse model of AR reported that mice treated with xyloglucan spray exhibited fewer symptoms, such as rubbing and sneezing, similar to those treated with cromoglycate and cetirizine [2]. Additionally, combining xyloglucan spray with antihistamines resulted in symptom relief comparable to budesonide nasal spray [2].

The safety profile of xyloglucan nasal spray was favorable, with no significant differences in adverse events between the treatment and placebo groups. Most adverse events were mild and comparable between the 2 groups, and no serious adverse events or study drug withdrawals occurred. This result is also in line with a study in adults [8]. This finding underscores the safety and tolerability of xyloglucan as a treatment option for pediatric AR.

To our knowledge, this study is the first prospective randomized, double-blind, placebo-controlled crossover study to evaluate the efficacy of xyloglucan nasal spray in controlling symptoms of AR in aeroallergen-sensitized children. However, our study has some limitations, including being a single-center study with a relatively small sample size. Additionally, the study duration was relatively short, which limits the assessment of the long-term safety of xyloglucan nasal spray. These limitations necessitate further multicenter studies with larger populations to validate our findings and explore xyloglucan’s long-term benefits and safety in managing pediatric AR. Despite these limitations, our study has several strengths. The randomized, double-blind, placebo-controlled crossover design minimizes bias and allows each participant to serve as their own control, enhancing the reliability of the results. The use of VAS provides a real-life assessment of the treatment’s efficacy. The lack of serious adverse events further supports the feasibility and safety of xyloglucan nasal spray in a pediatric population.

## 5. Conclusion

Our study provides evidence supporting the potential role of xyloglucan nasal spray as an adjunctive therapy to standard treatment for children with AR. Future research should focus on confirming these results in larger and more diverse pediatric populations and investigating the long-term efficacy and safety of xyloglucan nasal spray in treating AR.

## Conflicts of interest

The authors have no financial conflicts of interest.

## Author contributions

Piyapong Laopakorn, Wacharoot Kanchongkittiphon, and Wiparat Manuyakorn are involved in the conception and design. Piyapong Laopakorn, Adithep Sawatchai, Wanlapa Jotikasthira, Potjanee Kiewngam, and Wiparat Manuyakorn are involved in collecting and analyzing the data. Piyapong Laopakorn and Wiparat Manuyakorn are involved in the interpretation of the data. Piyapong Laopakorn drafted the manuscript. Wiparat Manuyakorn and Wacharoot Kanchongkittiphon critically reviewed the manuscript and gave the intellectual content. Wiparat Manuyakorn revised the final manuscript. All authors approved the manuscript. All authors agreed to be accountable for all aspects of the work.

## Acknowledgements

This study was funded by the Research Fund, Faculty of Medicine, Ramathibodi Hospital, Mahidol University.

## Supplementary material

Supplementary Material 1 can be found via 10.5415/apallergy.2022.12.e38

Supplementary Material 1

Click here to view
